# Rhabdomyosarcoma in a child with nephrotic syndrome treated with cyclosporine: a case report with literature review

**DOI:** 10.1186/s12882-020-02136-6

**Published:** 2020-11-17

**Authors:** Huai-Chueh Gem Wu, Chao-Neng Cheng, Jiann-Shiuh Chen, Yuan-Yow Chiou

**Affiliations:** 1 Eudcation Center, National Cheng Kung University Hospital, Tainan, Taiwan; 2 Division of Pediatric Hematology, Department of Pediatrics, College of Medicine, National Cheng Kung University Hospital, Tainan, Taiwan; 3 Division of Pediatric Nephrology, Department of Pediatrics, National Cheng Kung University Hospital, Tainan, Taiwan; 4 Institute of Clinical Medicine, Medical College, National Cheng Kung University, 138 Sheng-Li Road, Tainan, 704 Taiwan

**Keywords:** Childhood nephrotic syndrome, Cyclosporine, Immunosuppressive therapy, Rhabdomyosarcoma, Case report

## Abstract

**Background:**

In patients with frequently relapsing nephrotic syndrome, immunosuppressive therapy such as cyclosporine are often required to maintain remission. Cyclosporine has been noted to have tumorgenesis effects. In this case report, we present a child with relapsing nephrotic syndrom developed a rhabdomyosarcoma on her tongue after adout 4 years of continual immunosuppressive therapy.

**Case presentation:**

A 2-year-old female child had nephrotic syndrome (urine protein-creatinine ratio 749.1 mg/mg; blood urea nitrogen 11 mg/dL; serum creatinine 0.3 mg/dL; and serum albumin 1.8 g/dL.) Proteinuria resolved on treatment with daily prednisolone for 4 weeks at the dose of 45 mg (2.5 mg/kg/day) but recurred with taper from 25 mg/day to 10 mg/day. At least five more episodes of relapse occurred within about a 3-year period. After the third relapse, she was treated with prednisolone and cyclosporine (at initial dose of 50 mg/day [1.7 mg/kg/day]) for immunosuppression. About 4 years after the diagnosis of nephrotic syndrome had been made, an embryonal rhabdomyosarcoma developed on her tongue. The cancer was treated with TPOG-RMS-LR protocol, with vincristine, actinomycin, and cyclophosphamide. Magnetic resonance imaging scan, performed about 3 years after the start of TPOG-RMS-LR therapy, revealed complete remission of the cancer.

**Conclusions:**

Although treatment with cyclosporine cannot be conclusively implicated as the cause the rhabdomyosarcoma in this patient, the association should prompt consideration of its use in the treatment of frequently relapsing nephrotic syndrome in children.

**Supplementary Information:**

The online version contains supplementary material available at 10.1186/s12882-020-02136-6.

## Background

Nephrotic syndrome is defined by nephrotic-range proteinuria (≥40 mg/m2/h or urine protein/creatinine ratio ≥ 200 mg/mL or 3+ protein on urine dipstick), hypoalbuminemia (< 25 g/L) and edema [1]. The reported incidence of idiopathic nephrotic syndrome is 1.15–16.9 per 100,000 children, varying by ethnicity and region [2]. The cause remains unknown, but the pathogenesis is thought to involve immune dysregulation, systemic circulating factors, or inherited structural abnormalities of the podocyte. The disease most often resolves spontaneously, but corticosteroids often are required. About 1–3% of children with nephrotic syndrome have frequent relapses or steroid-dependent nephrotic syndrome. Resistance to steroids or steroid toxicity often prompts administration of other immunosuppressive drugs, such as cyclosporine (CsA), cyclophosphamide, rituximab [3]. and others. Rhabdomyosarcoma (RMS) is the kind of soft tissue sarcoma (STS) and most common occurs in children and adolescents [4]. RMS is accounting for 4.5% of all children cancer cases and the incidence is around 6 cases per million per year [5]. However, the aetiology of RMS is still need to be identified. Although tumorigenesis risks have not been found in children with nephrotic syndrome treated with CsA, one of the most feared adverse effects of the drug is de novo cancers [6, 7]. Herein we describe a patient with frequently relapsing nephrotic syndrome who received a long course of CsA and developed a rhabdomyosarcoma (RMS) of the tongue.

## Case presentation

A 2-year-4-month-old girl was referred to our hospital about 1 month after the diagnosis of nephrotic syndrome with relapse had been made: urine protein-creatinine ratio markedly increased (749.1 mg/mg); blood urea nitrogen 11 mg/dL; serum creatinine 0.3 mg/dL; and serum albumin 1.8 g/dL. Proteinuria resolved on treatment with daily prednisolone for 4 weeks at the dose of 45 mg (2 mg/kg)/day) but recurred with taper from 25 mg/day to 10 mg/day (spot proteinuria ≥300 mg/dL protein).

A second relapse occurred near 3 months after the first when the daily dose of prednisolone was decreased and proteinuria resolved with prednisolone at 45 mg/day for nearly a week.

A third relapse occurred 3 months after the second when she had taken 30 mg prednisolone once every 2 days for 3 weeks (spot proteinuria > 500 mg/dL). CsA was added at a daily dose of 50 mg/day (1.7 mg/kg/day). Prednisolone was tapered over 3 months and discontinued after about 1 years of regular use. CsA was prescribed continuously, with the dose of 50 mg/day since no proteinuria was noted. After a year of CsA maintenance without steroid, she developed bilateral leg edema, massive proteinuria, and decreased urine output. The dose of CsA was increased to 75 mg/day (3 mg/kg/day) for 4 months. The proteinuria resolved within 2 weeks under the combination of prednisolone 45 mg/day and CsA 75 mg/day, tapered to 50 mg/day for another 3 months.

The fifth relapse, 9 months from the previous relapse, occurred (spot proteinuria 100 mg/dl) after she had been prednisolone free for nearly 20 days. Since the fifth replace, the prednisolone was held at dose 60 mg/day and cyclosporine was prescribed with dose 100 mg/day.

Before CsA was replaced with mycophenolic acid 720 mg/day (20 mg/kg). The sixth relapse has occurred since daily dose of steroid was discontinued, and the proteinuria was controled by undertake prednisolone 45 mg/day. She has had frequent urinary tract infections during the four-year use of steroids with other steroid-sparing immunosuppressive therapy.

**Fig. 1:**
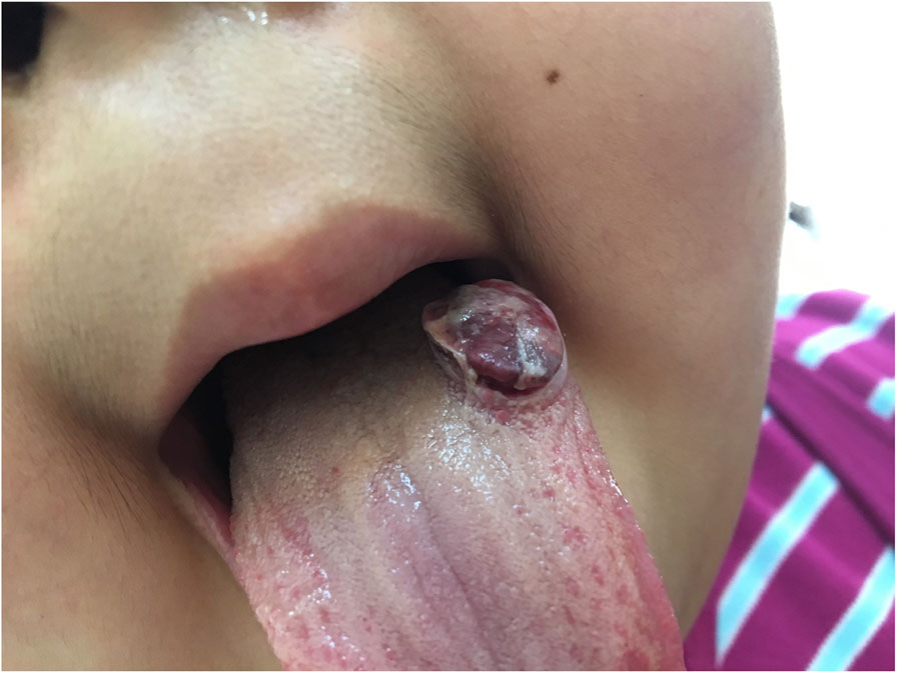
A reddish flat-topped mass on the tongue

At about 4 years after the diagnosis of nephrotic syndrome was made, reddish flat-topped masses appeared her tongue (Fig. 1). Curative wedge resection revealed an exophytic polypoid lesion that was histopathologically diagnosed embryonal RMS. After total excision of the tumor, she was treated according to TPOG-RMS-LR protocol, with vincristine, actinomycin, and cyclophosphamide (VAC). Mycophenolic acid and prednisolone were discontinued since the dose and duration of cyclophosphamide per TPOG protocol was more than the required amount for treatment of nephrotic syndrome. No proteinuria or nephrotic syndrome were noted during the 48-week VAC treatment and 10 months thereafter. The length and cumulative dosage of these immunosuppressive drugs are illustrated as supplemental figure 1.

## Discussion and conclusions

We know of no other reports of RMS in children treated with CsA for nephrotic syndrome, although a case of embryonal RMS in pediatric nephrotic syndrome without evident immunosuppressive therapy has been described [8]. CsA has been widely accepted in the treatment of frequent relapsing nephrotic syndrome in children [9, 10]. It is reported effective and safe in treatment of that condition to avoid the toxicity of corticosteroids [10]. Nevertheless, because of cyclosporine’s risk in a wide variety of cancers, including RMS [6, 7, 11–13], even an isolated case of RMS in a child with nephrotic syndrome should prompt caution about its use in that population.

CsA may promote tumor angiogenesis through VEGF and blockake T lymphocytes function under high-dose CsA (≥4–5 mg/kg/day) treatment [14]. The present study expanded this finding and further showed that CsA-related RMS has been described only in case reports (Table 1). Cescon et al. [11] reported a 23-year-old girl who received CsA for liver transplantation immunosuppression and later developed orbital embryonal RMS. A 47-year-old Japanese man, who received CsA for treatment of Behçet’s disease, developed a malignant rhabdoid tumor in his posterior femoral region 3 years later [12]. A 15-year-old boy, treated with CsA to prevent kidney transplant rejection, developed RMS of the nasopharynx [13]. The tumors of our patient and the patient of Cescon et al. [11] were of the embryonal histology, which is the most common subtype. Mycophenolate mofetil (MMF), an ester prodrug of mycophenolic acid (MPA), is known as an FDA-approved immunosuppression agent and exists the function of anti-tumor activity, which mainly acts on inosine monophosphate dehydrogenase (IMDPH) to treat with immune-related adverse events effectively [15, 16]. Thus, utilizing CsA may exert the risk in promoting tumorigenesis during the treatment course of disease.

**Table 1:** Case reports on cyclosporine associated rhabdomyosarcoma or rhabdoid tumor

*Year, Author*	*Patient data*	*Indication for CsA*	*Dosage (mg/kg/day)*	*Duration of CsA*	*Exposure to Diagnosis*	*Tumor type, location*	*RMS treatment*	*treatment response*
*Current case*	8 y/o female	nephrotic syndrome	1.8–3.0	3 years	4 years 2 months	embryonal, tongue	Resection VAC (48 weeks)	Complete remission (27 months)
*2003, Cescon* [11]	23 y/o female	liver transplant	13.0	16 months	16 months	embryonal, orbit	RT▲ VAC (24 weeks)	Stable Disease (38 months)
*1998, Muramatsu* [12]	47 y/o male	Behçet’s disease	5.0	9 months▽	3 years	malignant rhabdoid tumor, femur	Resection RT	Complete remission (36 months)
*1991, Piller* [13]	15 y/o male	renal transplant	N/A	23 months	23 months	rhabdomyosarcoma, nasopharynx	N/A	N/A

▲ 5000 cGy in 5 weeks

▽ After 9 months of cyclosporine, the patient found a mass over his posterior thigh. However, he did not seek medical attention until 3 years later

*CSA* Cyclosporine A, *RMS* Rhabdomyosarcoma, *RT* Radiation therapy, *VAC* Vincristine, actinomycin, and cyclophosphamide

Although over 95% of RMSs arise de novo, the role of immunosuppressants in their causation-- especially in transplant patients, who require high dose and life-long administration -- is persuasive. Further evidence of cyclosporine’s oncogenic potential is that patients receiving reduced-dose of drug have had a lower risk of malignancies than did those receiving full-dose [17]. There is no solid evidence supporting an oncogenic effect of CsA in pediatric RMS, but other carcinogenic factors have not been identified. Molecular mechanisms of cyclosporine’s tumor- promoting activity are incompletely defined. However, in human studies, CsA induced cancer progression via increasing production of TGF-beta and inhibiting T-lymphocyte function, and, in a murine model, T-cell-based therapy was effective in treating RMS [18]. These results suggest that defective T-cell immunity contributes to rhabdomyosarcoma oncogenesis. CsA also has caused defective nucleotide excision repair in cells [19]. However, RMS has been thought as the dominant hereditary genetic diseases [20], but some risk factors may trigger the occurrence of RMS including, the familial syndromes (Li-Fraumeni syndrome), the sign with a lump or swelling and keep getting bigger, and bulging eyes or hematuria. Otherwise, lifestyle-related risk factors, for examples, body weight, diet and physical activity, may not play a role in the occurrence of RMS, which is commonly occurred in childhood (under 10 years old). Altogether, the present case report suggests that the risk, albeit low, of rhabdomyosarcoma with cyclosporine immunosuppression be considered in the selection of immunotherapeutic agents in the treatment of relapsing nephrotic syndrome in children.

## Supplementary Information

**Additional file 1:** **Figure S1.** Schematic the time line of different drugs utilized in treatment of nephrotic syndrome patient with RSM.

## Data Availability

The data used to support the findings of this study are included within the article.

## Abbreviations

RMS: Rhabdomyosarcoma
CsA: Cyclosporine
TPOG-RMS-LR: Taiwan Pediatric Oncology Group-RMS-Low risk group
VAC: Vincristine, actinomycin, and cyclophosphamide
VEGF: Vascular endothelial growth factor
