# Supplementary material for: Rhabdomyosarcoma in a child with nephrotic syndrome treated with cyclosporine: a case report with literature review
Source: BMC Nephrol. 2020 Nov 17;21:490. doi: 10.1186/s12882-020-02136-6 (PMC7673093; doi:10.1186/s12882-020-02136-6)

**Supplemental figure 1.** Schematic the time line of different drugs utilized in treatment of nephrotic syndrome patient with RSM.


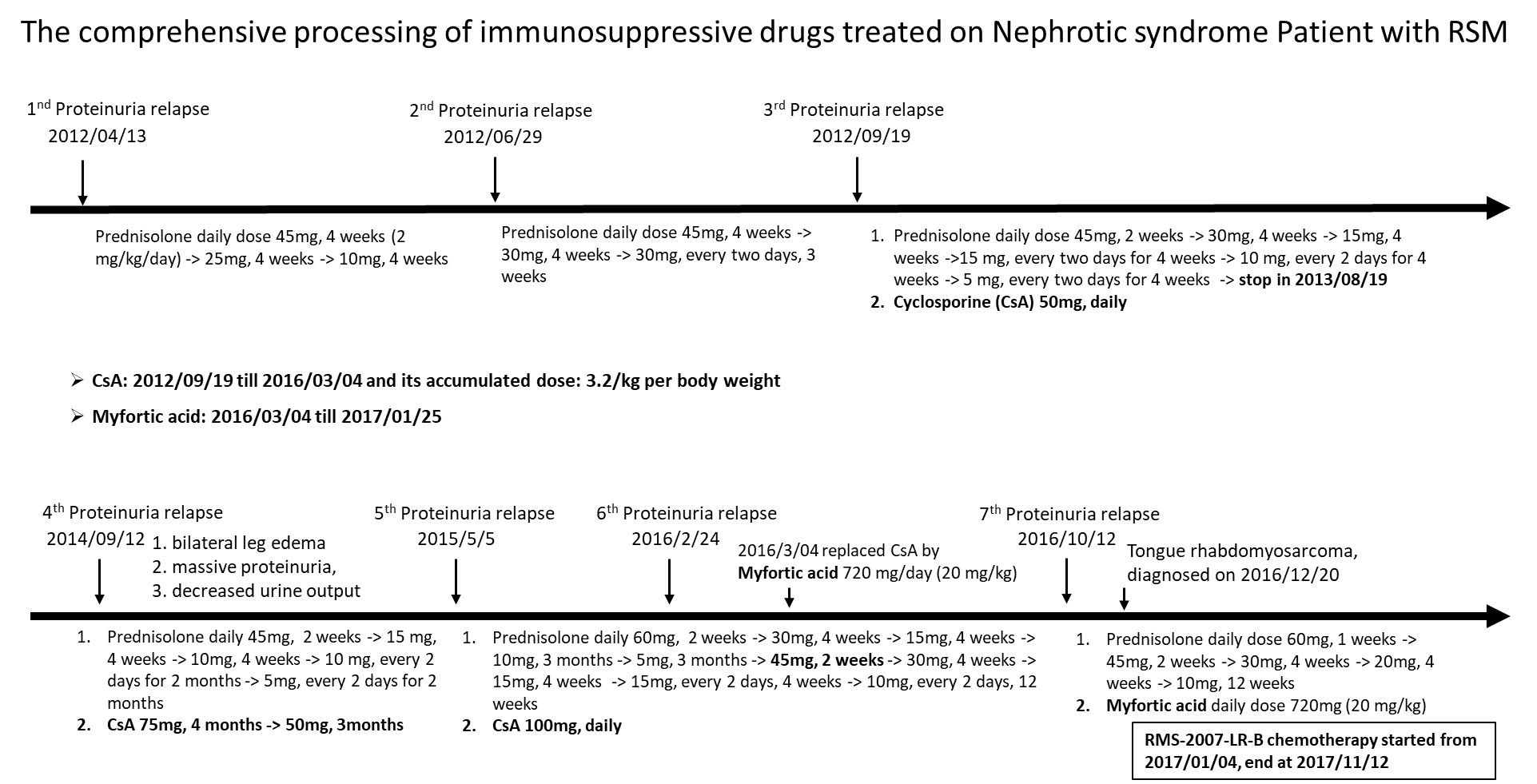

Supplement: Supplementary file 1 — Additional file 1: Figure S1. Schematic the time line of different drugs utilized in treatment of nephrotic syndrome patient with RSM. [file 12882_2020_2136_MOESM1_ESM.docx]
